# Comparison of the Influence of Polypropylene (PP) or Polybutylene Terephthalate (PBT)-Based Meltblown and Polyester/Polyamide-Based Hydroentangled Inner Layers on the Sound and Thermal Insulation Properties of Layered Nonwoven Composite Structures

**DOI:** 10.3390/polym17010101

**Published:** 2025-01-02

**Authors:** Emel Çinçik, Süreyya Kocatepe, Eda Aslan

**Affiliations:** 1 Department of Textile Engineering, Faculty of Engineering, Erciyes University, 38039 Kayseri, Türkiye; 2 Department of Textile, Clothing, Shoes and Leather, Firat University, 23300 Elazığ, Türkiye

**Keywords:** sound insulation, heat insulation, meltblown nonwovens, hydroentangled nonwovens

## Abstract

Thermal and sound insulation play a vital role in today’s world, and nonwoven composite structures including microfiber layers provide efficient solutions for addressing these demands. In this study, the sound and thermal insulation properties of nonwoven composite structures, including single-layer meltblown, multilayer meltblown, hydroentangled, and nanofiber nonwoven inner layers, were compared statistically by using Design Expert 13 software. The inner layer type and outer layer type of the composite structures were considered as independent variables, and thickness, bulk density, air permeability, sound absorption coefficient, and thermal resistance of composite structures were evaluated as dependent variables during statistical analyses. The effects of layer types on dependent variables were investigated comparatively, and the best inner and outer layers for high sound and thermal insulation were determined. It was concluded that the developed nonwoven composites including hydroentangled and three-layered meltblown layers demonstrated superior sound absorption properties at low (changing between 48% and 70%) and moderate (ranging between 77% and 96%) sound frequencies, respectively, when compared to composites and materials including single-layer meltblown or nanofiber nonwoven structures reported in prior studies. Additionally, it can be inferred that the composite structures obtained in this study exhibited thermal resistance properties (0.49 to 0.73 m^2^K/W) comparable to those of commercial thermal insulation materials.

## 1. Introduction

The rapid advancements in technology and the evolving demands of contemporary life in recent years have caused noise pollution to become a significant environmental issue, impacting human health and comfort. Unwanted sounds across a range of frequencies, commonly known as noise, can dull the senses, hinder focus, disrupt sleep, and cause irritation. Furthermore, extended exposure to high levels of noise can result in health issues such as tinnitus, hearing loss, neurological disorders, and high blood pressure. Consequently, noise control measures are crucial in sectors like construction, automotive, and manufacturing to enhance the quality of life [1,2,3,4,5,6].

Among the numerous techniques for noise control, sound insulation stands out as the favored approach. The textile-based fibrous sound insulators are widely preferred for blocking the spread of sound waves and reducing sound transmission. Nonwoven materials present notably advantageous solutions among textiles for sound absorption because of their intricate internal structure, low weight, affordability, and potential for recycling [4,5,6,7].

The nonwoven structures consist of fibers of varying lengths, along with cavities, channels, or spaces and pores that allow sound waves to penetrate the material when subjected to sound pressure. The air molecules inside the pores oscillate at the frequency of the incoming sound wave, which results in frictional losses that convert sound energy into heat. Additionally, the air molecules within the pores experience rhythmic compression and relaxation from sound waves, causing temperature changes. At low frequencies, heat exchange occurs isothermally due to prolonged exposure, large surface-to-volume ratios, and the high heat conductivity of fibers. At high frequencies, compression is adiabatic. Between these frequency regions, heat exchange leads to the loss of original sound energy. Also, momentum loss occurs with the alteration in the direction of sound wave flow, combined with the expansion and contraction within the irregular pores. Moreover, the interlocked fibers within nonwovens serve as frictional elements, resisting the motion of acoustic waves. As a result of individual fiber vibration, a substantial amount of sound energy is absorbed through scattering by the fibers. Briefly, the intensity of sound energy diminishes due to momentum dissipation, frictional resistance, and temperature fluctuations occurring within the complex and porous framework of nonwoven materials [2,7,8,9,10,11,12,13].

The detailed literature review from our previous study [14] revealed that several crucial factors such as areal weight, thickness, bulk density, pore size, pore shape, and pore quantity of the nonwovens significantly influence the sound-absorbing characteristics of the structure. Since the properties of the fibers that compose the nonwoven materials, including type, elasticity, length, fineness, and cross-section, and structural variables like fiber orientation and number of layers influence the aforementioned characteristics, these parameters have also been found to have an effect on the sound absorption capabilities of nonwoven materials [2,5,7,11,12,13,14,15,16]. Furthermore, earlier investigations revealed that although conventional nonwoven structures presented high sound absorption characteristics at higher sound frequencies, lower and inadequate sound absorption coefficients were obtained for low and moderate frequencies reaching 2 kHz [16,17,18,19,20,21,22,23].

Enhancing sound absorption in low-frequency bands can be achieved through several methods: using nonwovens with greater thickness, increasing the depth of the backing air cavity, or decreasing the fiber fineness. The latter method was said to be more effective due to the area restrictions of other methods. To maintain the same volume density with finer denier fibers, a larger number of fibers is needed, leading to a more complex pathway and greater resistance to airflow. Furthermore, reducing fiber diameter leads to an increase in surface area and an increase in friction between pore surfaces. Also, fibers with lower diameters are more responsive to sound waves than thicker ones, and the structures containing finer-diameter fibers act as acoustic resonance membranes at low and medium sound frequencies. Therefore, sound absorbers that include nanofibers [24,25,26,27,28,29,30,31,32,33,34,35,36,37,38,39,40,41,42,43,44,45,46,47,48,49,50] and microfibers [7,11,14,17,28,51,52] in earlier studies have been found to be more beneficial.

As highlighted in our former research, nonwovens comprising microfibers are advantageous compared to nanofiber webs in terms of simplicity of production and manufacturing time, and sound absorbers encompassing microfibers can be practical substitute for nanofibers. These surfaces can be produced using three methods: microfiber utilization in the carding process, meltblown method, or hydroentangling method. The microfibrous surfaces obtained by carding are bulkier than those produced by electrospinning, thus they do not exhibit a membrane structure and are considered ineffective for low- and medium-frequency sound insulation. In our previous study, the influence of raw material, areal weight, and process conditions of single-layer meltblown nonwoven inner layers on the thermal and sound insulation characteristics of nonwoven composite structures was evaluated, and it was indicated that single-layer meltblown-incorporated composite structures demonstrated equivalent or superior sound absorption values compared to a nanofiber layer, including counterparts and structures evaluated in early studies [14].

Nonwovens with hydroentangled microfibers are produced from spunbond nonwoven surfaces made from endless filaments with a segmented pie cross-section. These structures are subjected to water jets with high pressure, and the filaments in the material are split into microfilaments and entangled thanks to the high-pressure water jets [53]. Microfibrous hydroentangled and meltblown nonwovens can be produced cost-effectively, resulting in lighter nonwoven fabrics characterized by smaller fiber and pore diameters, along with an expanded surface area. It was determined that only a small number of earlier studies [54] dealt with the sound insulation characteristics of hydroentangled nonwovens. Furthermore, limited numbers of investigations have been conducted on the thermal insulation properties of hydroentangled [53,55,56] or meltblown nonwoven layers containing composite structures.

This study makes a unique contribution to the literature by comparing composite structures containing single-layer meltblown, three-layer meltblown (MMM), and hydroentangled microfibrous nonwoven inner layers, as well as their nanolayer counterparts. For this purpose, and as a continuation of our previous study, composite structures containing single meltblown layers and exhibiting the best sound absorption properties for different sound frequency bands were selected from our earlier study [14]. These were then compared with composites constructed using hydroentangled and three-layered meltblown microfibrous nonwovens with the same areal weight and nanofibrous inner layers. Different types of single-layer meltblown nonwovens, including calendered and non-calendered variations made from different raw materials (polypropylene and polybutylene terephthalate), as well as hydroentangled and three-layered (MMM) meltblown nonwovens, were used as inner layers with the same areal weights. Thermo-bonded outer layers, manufactured with three different fibers [14], were combined with these inner layers to form the composites. The physical properties (thickness, bulk density) as well as performance features (air permeability, sound absorption coefficient, and thermal resistance) of the composites were tested, and the results were statistically analyzed using Design Expert 13 software by considering outer and inner layer type as independent variables. It was concluded that hydroentangled nonwoven inner layers can be an alternative to single meltblown or nanofiber nonwovens at low sound frequency bands, whereas three-layered meltblown nonwovens can be at mid frequencies. Additionally, non-calendered single meltblown and nanofiber inner layers maintained their leadership in terms of sound reduction at high sound frequency levels compared to hydroentangled or three-layered nonwoven counterparts.

## 2. Materials and Methods

### 2.1. Materials

**Figure 1:**
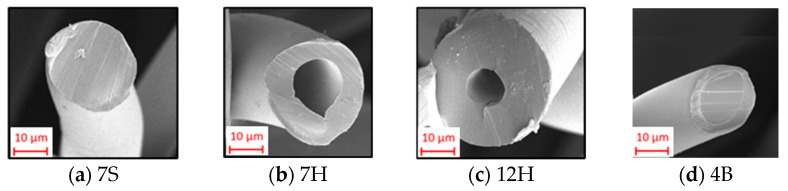
The fibers constituting the thermally bonded outer layer.

The nonwoven composite structures composed of two thermally bonded nonwoven outer layers and one inner layer were formed to develop a unified material that can accommodate various sound frequencies, as specified in our previous study. This study differs from the other research by incorporating hydroentangled and three-layered meltblown layers with the same areal weight in addition to single-layer meltblown layers as inner layers. Recycled polyester (r-PET) fibers with different cross-sectional shapes and diameters were used to produce outer layers: 7 deniers with solid cross-section (Figure 1a), and 7 and 12 deniers with hollow cross-section (Figure 1b,c). Also, 4 denier polyester/co-polyester bicomponent fiber (Figure 1d) was used to form bonds with the other fibers. The characteristics of the fibers that make up the outer layer were thoroughly outlined in our earlier study [14].

### 2.2. Methods

#### 2.2.1. Individual Layers and Composite Production

All the inner layers composed of microfibers were produced using two methods: meltblown and hydroentangling method at a constant areal weight of 200 g/m^2^, as demonstrated in Table 1. The single layer of meltblown inner layers (M coded) was manufactured from either Polypropylene (PP) or Polybutylene Terephthalate (PBT) as raw material, and layers were either calendered (C) or non-calendered (NC). Further specifics on this topic are available in our prior research [14]. The hydroentangled inner layer (H coded) was formed by applying water jets with high pressure on segmented pie cross-sectional spunbond nonwoven made of 70% polyester /30% polyamide. Three-layered meltblown structures (MMM coded) were constructed by assembling and calendering three polypropylene-based meltblown layers with an areal weight of 30-140-30 g/m^2^ in a way such that the areal weight remained the same as that of other inner layers. The calendering process for the MMM layer was accomplished by using 5 mm circular engraved cylinders at 160 °C. In addition to inner layers containing microfibers, electrospun nanofiber webbing formed from polyamide was alternatively used as inner layers for comparison purposes.

As mentioned in our early study, the blend ratio of the outer layer was applied as 80% main fiber (7S, 7H and 12H) and 20% polyester/co-polyester bicomponent fiber (4B). The carding, cross-lapping, and through-air-bonding process steps were followed to produce this layer, and extensive details about these processes can be found in our previous work [14].

Three-layered nonwoven composites were constructed by using bulky thermally bonded layers as external layers and microfiber/nanofiber layers as internal layers. Twenty-one different three-layered composite structures were formed by combining three different outer and seven different inner layers (Table 2). The details concerning the manufacturing processes of composites are available in our prior research [14].

#### 2.2.2. Tests Conducted on Individual Layers and Composites

The composite structures and individual external and internal layers were conditioned for 24 h under standard atmospheric conditions as specified by the ISO 139 prior testing procedure [57], and all tests were carried out in a controlled environment with a temperature of 20 ± 2 °C and humidity of 65 ± 4%. The weights per unit area of the structures and individual layers were determined following test standard NWSP 130.1 [58] by evaluating 10 samples of 30 cm x 30 cm each. The thickness of the inner layers was measured using a Digital Thickness Gauge (Elastocon EV 07, Elastocon AB, Brämhult, Sweden) according to NWSP 120.1 [59]. Alternatively, because of voluminous structure and sensitivity to pressure, the thicknesses of single outer layers and the composite structures were measured with digital calipers. The bulk density (*d_n_*, g/cm^3^) and porosity of the samples were calculated by using equations indicated in our prior study [14]. The densities used in the calculations for r-PET, bicomponent PET, PET, PP, PBT, and Polyamide were 1.35 g/cm^3^, 1.38 g/cm^3^, 1.38 g/cm^3^, 0.9 g/cm^3^, 1.35 g/cm^3^, and 1.14 g/cm^3^, respectively [60,61,62,63,64,65]. Moreover, because of the varying structural parameters of microfibrous and nanofibrous layers, the pore size characteristics of the meltblown, hydroentangled, and nanoweb layers were evaluated following the ASTM E1294 test standard, using a Capillary Flow Porometer (PMI, New York, NY, USA) [66].

The samples were subjected to air permeability tests with a Digital Air Permeability tester (Textest FX 3300, Switzerland) in accordance with the NWSP 070.1 [67] test standard employing a 20 cm^2^ test area and 200 Pa pressure differential. A heat flow meter (Thermtest HFM-100, Canada) was used to measure the thermal conductivity of the samples by following the ASTM C518 test procedure [68], and then the thermal conductivity values of the samples were converted to thermal resistance values (R: m^2^K/W) [14].

An impedance tube (Brüel&Kjær 4206 Model, Brüel & Kjær Sound &Vibration Measurement, Naerum, Denmark) was employed for assessment of the sound absorption coefficients of samples between 50 Hz and 6300 Hz sound frequency intervals, and the ISO 10534-2 and ASTM E1050–08 standards were applied during the tests [51,69,70]. The measurements for frequencies between 50 Hz and 1600 Hz were conducted using a large tube with a 100 mm diameter; on the other hand, a smaller tube with a 29 mm diameter was used for sound frequencies changing between 1600 Hz and 6300 Hz.

#### 2.2.3. Application of Statistical Analysis

The results from the tests were statistically evaluated through Design Expert 13 software by using a general factorial design. In the analysis, the types of external layers (O; 7DS, 7DH, 12DH) and internal layers (I; M-PP-C, M-PP-NC, M-PBT-C, MPBT-NC, H, MMM, N) were selected as independent variables. Furthermore, the sound frequency range (F; 50–6300 Hz) was included as an additional independent variable when analyzing the sound absorption coefficient. The influence of independent variables on dependent variables such as the thickness, bulk density, air permeability, thermal resistance, and sound absorption coefficient was utilized by investigating the analysis of variance (ANOVA) tables of each composite property. Consequently, the sound and thermal insulation properties of constructed composites were compared, and the inner and outer layer types that yielded the highest outputs were identified via the graphs generated by the software.

## 3. Results and Discussion

### 3.1. Layer Features

The physical characteristics (areal weight, thickness, bulk density, porosity) and air permeability of the individual outer layers were thoroughly examined in our previous study [14]. The areal weight of outer layers was approximately 200 g/m^2^ for all outer layers composed of different fibers. When the thickness of all the outer layers was evaluated, the layer composed of 7 denier hollow fiber (7H) had the highest thickness, followed by those including 7 denier solid fiber (7S) and 12 denier hollow fiber (12H). Additionally; the highest air permeability was provided by the outer layer containing 12 denier hollow fibers owing to the lager fiber diameter and lowest nonwoven thickness. In terms of air permeability, the outer layer with 12 denier fiber was followed by layers comprising 7 denier hollow and 7 denier solid fiber, respectively. Despite the higher thickness of the 7DH fiber-containing outer layer, it was assumed that the hollow fiber structure resulted in a looser and more porous surface, which contributes to greater air permeability compared to the 7DS fiber-involved outer layers.

Table 3 outlines the features of microfibrous layers and nanolayer used as internal layers in this study. As indicated in the table, all the microfibrous layers had an areal weight of about 200 g/m^2^ and thicknesses ranging between 0.53 and 2.40 mm. The hydroentangled layer displayed the lowest thickness, while the three-layered meltblown layer had the highest thickness of all. Moreover, as observed in the same table, single-layer meltblown inner layers made from polypropylene (PP) exhibited greater thickness compared to those fabricated from polybutylene terephthalate (PBT). This difference arises from the lower density and distinct structural characteristics of the PP polymer [61,64]. Due to the lower density of PP, a higher number of fibers was required to produce meltblown nonwovens with an equivalent areal weight, resulting in a bulkier and thicker structure compared to PBT-based single-layer meltblown nonwovens. Regarding the evaluation of more compact inner layers such as calendered and hydroentangled inner layers, the PP-based calendered meltblown surface showed the highest thickness due to its lower fiber density, followed by the calendered PBT-based structure and the hydroentangled Polyester/Polyamide-based layer. Although the overall fiber density of the hydroentangled inner layer was lower than that of the PBT-containing calendered inner layer, a thinner structure was obtained with hydroentangled layers. This result was considered to be due to the more compacting effect of the hydroentangling process compared to the calendering process.

When evaluating pore size, it was observed that, as expected, the nanolayer had the smallest pore size, followed in order by the calendered PP-based single-layer meltblown, the hydroentangled, and then three-layered meltblown (MMM) layer. The lowest air permeability was observed for hydroentangled, single-layer calendered polypropylene-based meltblown, single-layer calendered polybutylene terephthalate-based meltblown, and three-layered meltblown layers, respectively.

### 3.2. Composite Structure Features

#### 3.2.1. Thickness and Bulk Density

Thickness and bulk density in nonwovens are key for analyzing structural properties and understanding the connection between structure and performance. Table 4 provides a summarized analysis of variance (ANOVA) for thickness and bulk density values for the generated nonwoven composite structures. Here, “O” denotes the type of the outer layer while “I” represents the type of inner layer.

The table indicates that model parameters with *p*-values lower than 0.05 had a statistically significant impact on thickness and bulk density at the 95% confidence level. A significant effect means that the outer layer type and inner layer type led to a statistically meaningful change in the thickness and bulk density of the composite. The factor or model contribution is calculated by dividing its sum of squares by the sum of squares of the corrected total. The model’s contribution, or R^2^ (coefficient of determination), is a statistical indicator that represents the proportion of change in the dependent variable clarified by the independent variables. The generated models showed an R^2^ of 96.33% for thickness and 98.89% for bulk density, which implies that the selected factors explain 96.33% of the alteration in thickness and 98.89% of the alteration in bulk density.

As seen from the table, the linear effects of outer layer and inner layer type had significant effects on both the thickness and bulk density of the composites. The higher F values suggest greater effects and greater contribution of the factors on composite properties. As can be inferred from the figure, the effect of the outer layer type was found to be greater than the effect of the inner when F values were considered. This is believed to be because of the very thin nature of the inner layers, resulting in minimal impact on the overall thickness and bulk density.

**Figure 2:**
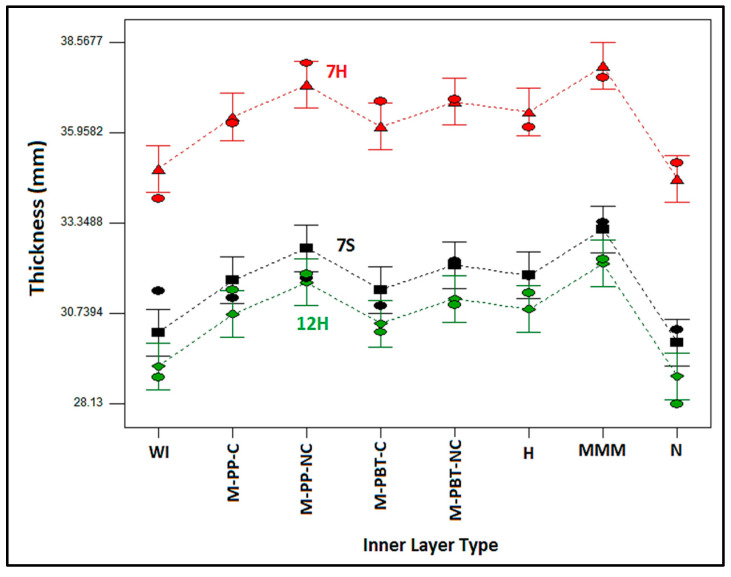
Variation in composite thickness with internal and external layer type.

**Figure 3:**
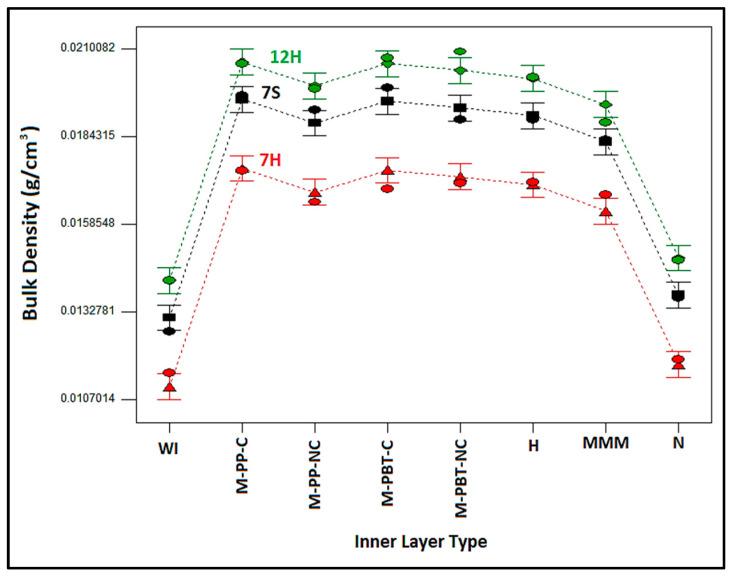
Variation in composite bulk density with internal and external layer type.

The influences of the outer and inner layer types on the thickness and bulk density of nonwoven composite structures are demonstrated in Figure 2 and Figure 3, respectively. Upon comparing the composites with various outer layers, as depicted in Figure 2, it was deduced that the minimum thickness was obtained in composites with the hollow cross-sectional 12 denier fiber-containing outer layer (12H), followed sequentially by those that had solid cross-sectional 7 denier (7S) fiber and hollow cross-sectional 7 denier fiber-containing (7H) external layer. A similar thickness trend was observed for the individual outer layers [14], suggesting that these thickness measurements were reflected in the composites.

Regarding the inner layer types of the composites, it was observed that the thickness of composites also followed the trend of the individual inner layer thickness (Table 3). As illustrated in Figure 2, it was determined that composites with the MMM inner layer exhibited the greatest thickness, followed in order by composites with non-calendered PP and non-calendered PBT-based inner layers. It was considered that the MMM inner layer was found to be thicker due to the combination of three different meltblown layers, which consequently enhanced the thickness of the composites containing this inner layer. Non-calendered inner layers presented higher thicknesses than their calendered counterparts, as projected. The composites with inner layers containing nanofibers demonstrated the lowest thickness, similarly to the composites without inner layers (WI). As a final point, the hydroentangled meltblown inner layer-containing composites had the lowest thickness after their nanofiber-based inner layer counterparts.

Additionally, the inner layers fabricated from polypropylene (PP) polymer displayed greater thickness compared to those made from polybutylene terephthalate (PBT). This difference is attributed to the PP polymer’s lower density and structural characteristics [60,61]. The reduced density of PP necessitated the use of additional fibers to attain the same areal weight in the meltblown nonwoven fabric, resulting in a bulkier and thicker structure than that of PBT.

When the impact of inner and outer layer types on bulk density of the nonwoven composite structures (Figure 3) were evaluated, it could be concluded that similar bulk density results were obtained except for MMM, Nano inner layer-containing composites and composites without an inner layer (WI). The figure shows that the outer layer containing 12H fibers produced the densest structure, followed by nonwoven composite structures with outer layers containing 7S and 7H fibers, sequentially. Because the outer layer with 7H fibers had the highest thickness with equivalent areal weights, and the outer layer with 12H fibers had the lowest thickness, the bulk density of composites containing these outer layers was believed to be arranged in the single outer layer bulk density order [14]. It is proposed that the outer layer type, identified as the primary factor affecting bulk density in the ANOVA results (Table 4), imparted a similar pattern to the composite as seen in the bulk density of the individual outer layers.

Upon examining the change in bulk density of structures with different inner layers, the lowest density was acquired for composites without an inner layer (WI), as expected. Including more, denser inner layers (ranging from 0.088 g/cm^3^ to 0.255 g/cm^3^) between less-dense outer layers (varying from 0.0137 g/cm^3^ to 0.0173 g/cm^3^) [14] increased the overall bulk density of the composite. Nanofiber and MMM inner layer-containing composites presented the lowest bulk density values after composites without an inner layer. These results were attributed to the higher thickness of MMM layer samples and the special characteristics of the nanofiber layer. The highest bulk density results were obtained with calendered inner layers compared to those with non-calendered and hydroentangled ones. The calendering process includes the application of pressure and heat to the meltblown nonwoven surfaces to enhance bonding, leading to a denser, more compact, and tightly consolidated structure. Thus, this characteristic of the calendered inner layers is thought to be reflected in the composite structure. On the other hand, although the bulk density of the individual hydroentangled inner layer was the highest among the inner layers, composites containing single-layer meltblown inner layers exhibited slightly higher bulk density compared to composites with the hydroentangled inner layer. This situation was presumed to originate from higher regional variations inherent in the structure of the hydroentangled nonwoven layer caused by the different impact of water jet pressures on different regions (Table 1 and Table 3).

#### 3.2.2. Air Permeability

The air permeability of a textile structure is the measure of the ability of air to penetrate through the fibers and textile structure. Evaluating this property in composite structures was essential for comprehending the overall structure and porosity of layered nonwoven composite materials. As a result of statistical analysis on the air permeability of the generated nonwoven composites, the model identified as the most appropriate for characterizing the air permeability of layered nonwoven composite structures with varied inner and outer layers was found to be the 2FI model. Table 5 provides the ANOVA results for this model.

The influence of inner and outer layer types individually and the binary interaction of these two factors on air permeability were statistically significant, as depicted in the Table. Also, the generated model explained 99.9% of the variation in the air permeability of the nonwoven layered composites. The primary factor affecting the air permeability of the layered structure was identified as the inner layer, which contributed 98.16%. As discussed in the explanations related to bulk density, it was suggested that the denser inner layer controls the air permeability characteristics of the overall nonwoven composites.

**Figure 4:**
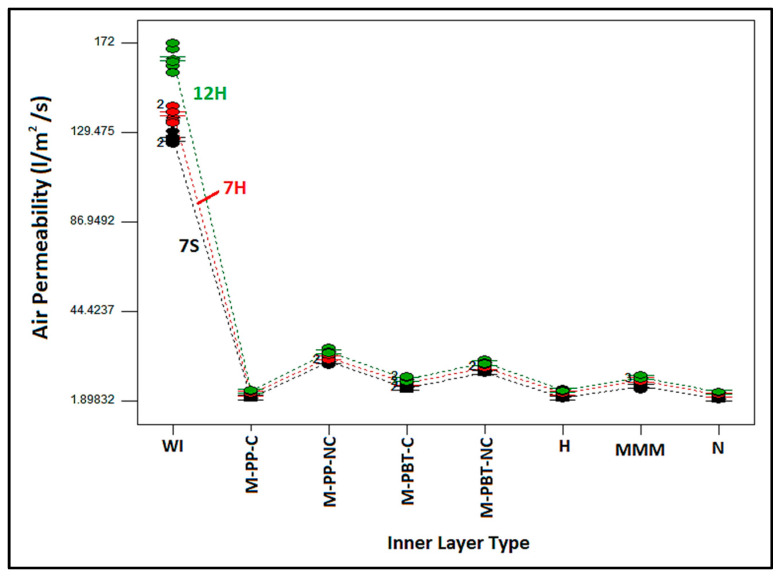
Relation between inner layer and outer layer type and air permeability of nonwoven composite structures.

**Figure 5:**
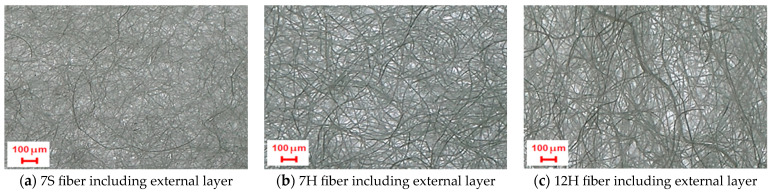
Surface visualization of external layers composed of various fibers (3×).

Figure 4 discusses the variation in air permeability of nonwoven composites with different outer and inner layers. It is evident that the air permeability of the composites without the inner layer (WI) had the highest air permeability values. This implies that the addition of an inner layer with a reduced pore size and an increased density led to a reduction in air permeability. Considering the air permeability results for composites with different outer layers, despite closer values, the lowest air permeability across all composites was observed in samples formed by an outer layer containing 7S fiber, followed by those with outer layers of 7H and 12H fiber composition. This phenomenon is more pronounced in composites that do not include an inner layer (WI). A parallel hierarchy was observed in the specific air permeability of each external layer [14], indicating that each layer feature also impacted the overall performance of the layered composite. Fewer fibers are required in the cross-section of nonwovens to achieve the same areal weight for 12H fiber-involved outer layers owing to the higher linear density of 12H fibers. This leads to larger and more voids, thereby facilitating air passage between the thicker fibers. The surface images of the outer layers are presented in Figure 5 to clarify this situation. Moreover, regarding the reduced thickness of this layer, it is projected that samples derived from this layer exhibited enhanced air permeability. Although the outer layer with 7DH was thicker, it was deduced that its hollow fiber structure creates a looser, more porous surface, enhancing air permeability in comparison to the outer layers composed of 7DS fiber.

It can be seen from Figure 4 that similar air permeability values were obtained although the composites were formed with different inner layers. Also, the ranking of air permeability for the individual inner layers was similarly reflected in the sandwich structures in all composite structures. In parallel with the individual inner layer values, the air permeability of the sandwich structures was highest in composites with non-calendered PP single meltblown inner layers, followed sequentially by non-calendered PBT single meltblown, three-layer meltblown (MMM), calendered PBT single meltblown, calendered PP single meltblown, hydroentangled, and nanofiber layers.

The primary factors affecting air permeability are pore size and thickness. Additionally, greater thickness, smaller pore size, and lower porosity tend to reduce air permeability [71]. The lowest air permeability results for composites containing nanofiber, PP-based calendered single meltblown, and hydroentangled layers were considered to be because of denser structures and smaller pore sizes. On the other hand, despite the much larger pore size of PP calendered meltblown and hydroentangled inner layers compared to nanofiber layer, it was estimated that the higher thickness of these layers led to similar air permeability results for nanofiber counterparts, and composites with these inner layers can be competitive with nanoweb-included composites. Also, as inferred from Figure 4, nonwoven composites with non-calendered inner layers exhibited the highest air permeability compared to composites with other inner layers due to the looser structure.

#### 3.2.3. Sound Absorption Coefficient

The sound absorption coefficient measures how effectively a material or surface reduces sound energy at a specific sound frequency. It reflects the proportion of incoming acoustic energy that is absorbed, as opposed to being reflected or transmitted. Typically, this coefficient is expressed as a dimensionless value between 0 and 1, where 0 signifies total sound reflection and 1 indicates total sound absorption. In this study, the sound absorption coefficients of nonwoven composites were determined at 19 distinct frequencies spanning from 100 to 6300 Hz. The recorded sound frequency levels (F) were included as an extra independent variable during statistical analyses to create a model for assessing the sound absorption characteristics of the nonwoven composites. Table 6 illustrates the variance analysis of the generated statistical model for the sound absorption coefficient of multilayered nonwoven composites at varying frequencies.

The R^2^ value of the specified model was found to be 95.83%, indicating that the outer layer type (O), inner layer type (I), and sound frequencies (F) account for 95.83% of the change in the sound absorption coefficient of the composites. It was observed that all selected factors had a notable impact on the sound absorption coefficient. The sound frequency was identified as the most dominant factor among them, contributing 58.24% to the sound-absorbing behavior.

**Figure 6:**
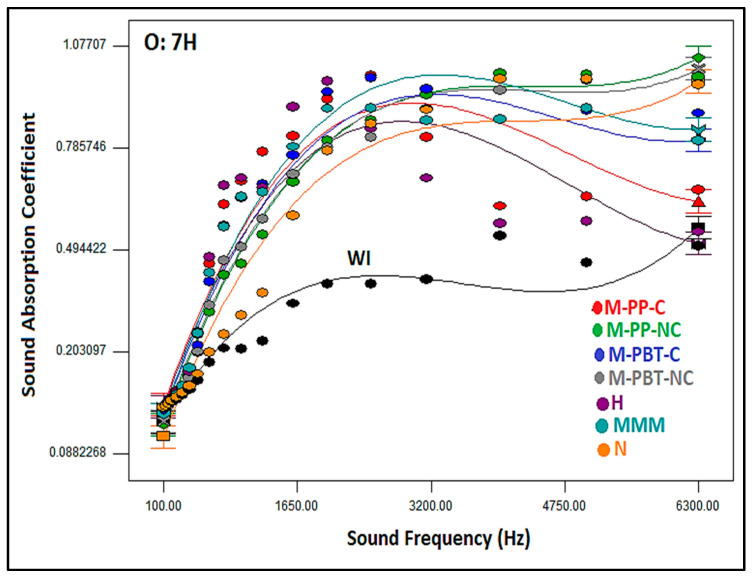
Relation between sound frequency and composite sound absorption coefficient by inner layer type, for composites including 7DH outer layer.

Figure 6 demonstrates the variation in the sound absorption coefficient of multilayered nonwoven composite structures concerning sound frequencies and inner layer type, specifically for the 7H fiber-integrated outer layer. Similar results were obtained with outer layer types comprising 7S and 12H fiber. As shown in the graph, the sound absorption coefficient increased with rising sound frequencies, and adding inner layers enhanced the sound absorption coefficient of the layered nonwoven composite without the inner layer (WI). The findings indicated that composites with hydroentangled, calendered, and MMM inner layers presented efficacy in low and moderate sound frequencies, whereas those including non-calendered and nanofiber inner layers were effective at higher frequencies. Particularly, composites with a hydroentangled inner layer exhibited the highest sound-absorbing behavior in the 630–2500 Hz frequency range. Additionally, the composites containing nanofibrous inner layers exhibited lower sound absorption coefficients at low and moderate sound frequencies compared to other counterparts, but similar results with calendered meltblown counterparts were obtained for higher sound frequency levels. The sound absorption coefficients reached peak values of 0.48 at 630 Hz, 0.71 at 800 Hz, and 0.74 at 1000 Hz, in sequential order. The findings for low frequencies surpassed the sound absorption coefficients documented in earlier studies on nanofiber layers [26,27,28,29,30,31,32,33,34,35,36,37,38,39,40,41,42,43,44,45,46,47,48,49,50]. Additionally, sound absorption values ranging from 0.77 to 0.96 were achieved for moderate frequencies (1250–3150 Hz), while values of 0.99 were attained for the upper-frequency range (4000–6300 Hz) with the composites in this research. Moreover, these values were considerably higher than those obtained in earlier studies [8,9,10,17,18,19,20,21,22,23,24,25,26,27,28,29,30,31,32,33,34,35,36,37,38,39,40,41,42,43,44,45,46,47,48,49,50,51,52].

Based on the developed model, the influence of the chosen inner and outer layer independent factors appeared to be negligible relative to the independent factor named frequency of sound. This was ascribed to the considerable variation in sound absorption across different sound frequencies, and it was believed that the impact of sound frequencies overshadowed the influence of the layer type parameters. To reveal the effect of the inner and outer layer types, sound frequencies in the 100–1000 Hz range were classified as low, sound frequencies in the 1250–3000 Hz range as medium, and sound frequencies in the 4000–6300 Hz range as high. The averages of the results within these ranges were then calculated and named as Noise Reduction Coefficient (NRC), as mentioned in previous studies [2,11,12,13,14]. Statistical analyses were repeated for NRC values at high, medium, and low frequencies by considering the NRC values as the dependent variable and excluding the sound frequency factor. The analysis of variance tables for the described analyses are summarized and presented in Table 7.

Upon assessing the table, the analysis revealed that the most effective models for characterizing the NRC for the composites across low, moderate, and upper-frequency sounds were identified as linear models including the outer layer and internal layer main factors (O, I). The R^2^ values for the models were found to be 94.33% for low frequencies, 97.61% for medium frequencies, and 97.37% for high frequencies. The results indicated that the most influential factor was the inner layer type, with 88.26%, 95.60%, and 96.63% contributions for low, medium, and high sound frequencies, respectively. As followed from the table, also the impact of the outer layer type was reduced by increasing sound frequency, in contrast to the inner layer type.

**Figure 7:**
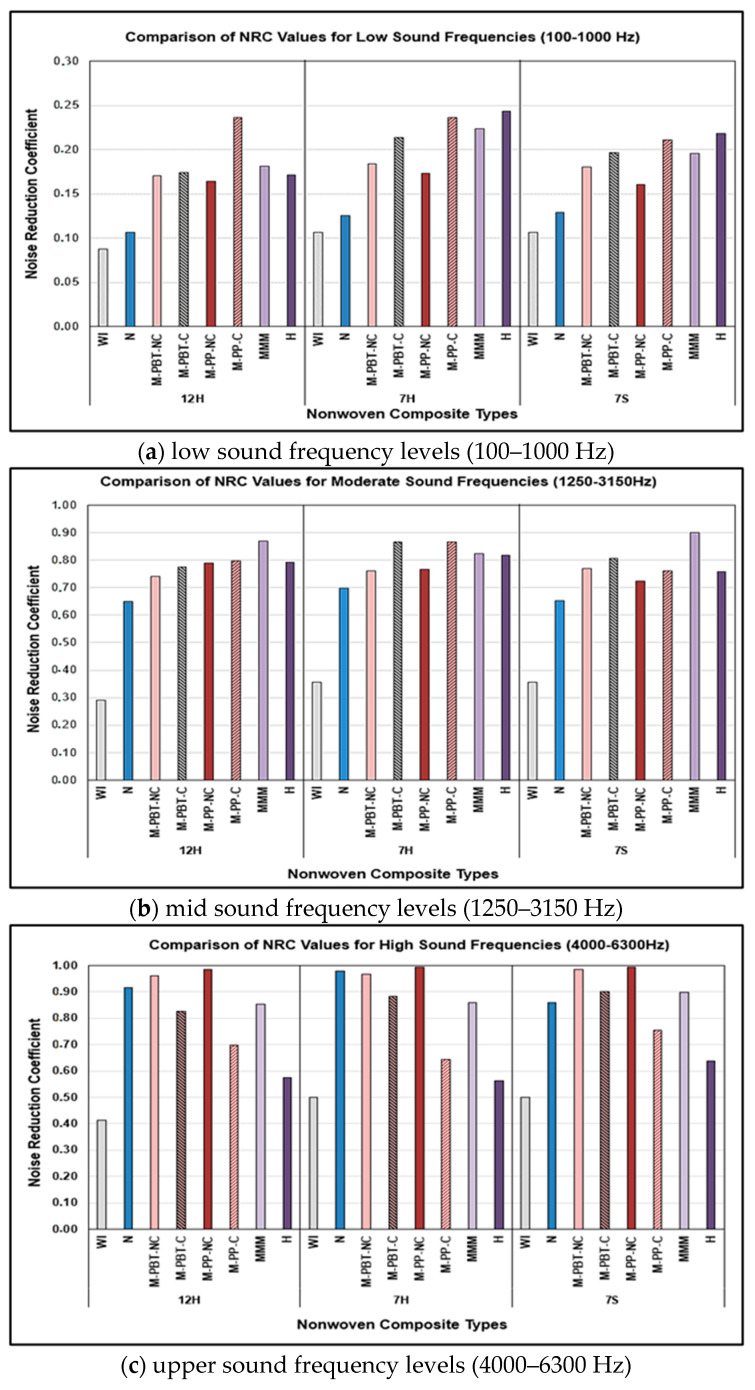
Analysis of noise reduction properties for various nonwoven composite structures across distinct sound frequency ranges.

Figure 7 illustrates the effects of outer layer type and inner layer type on the noise reduction coefficients for low (Figure 7a), medium (Figure 7b), and high (Figure 7c) sound frequencies. As demonstrated by the figures, despite approximately similar noise reduction coefficients, it was found that incorporating 7H fiber-containing outer layers into composite structures resulted in slightly better sound absorption in the lower frequency ranges. On the other hand, alteration of the outer layer type led to a negligible change in NRC at the mid and high frequencies for all nonwoven composites developed in this research. This situation can arise from the decreasing effect of the outer layer type by increasing sound frequency, as demonstrated in the variance analysis table (Table 7). By examining the table, it was observed that the outer layer’s individual contribution was higher for low frequencies (6.07%), but decreased to 2.01% for moderate and 0.74% for high sound frequencies (Table 7).

As mentioned in the introduction, using thicker nonwovens has been noted in previous studies as a method for enhancing sound absorption in low-frequency bands [2,8,24]. Because of the hollow fiber’s special characteristics and finer fiber structure, an outer layer incorporating 7H fiber provided both greater thickness and a more voluminous structure to the composites. Therefore, it was believed that 7H fiber-integrated composites presented better sound absorption values in the low sound frequencies. Additionally, the porosity value of the 7H fiber-containing outer layer was high compared to other outer layers [14] and also led to slightly better sound insulation. The findings suggest that an increased number of pores within the structure, as demonstrated in earlier research [2,8,9,10,11,12,13], enhanced the oscillation of air particles, thereby facilitating noise reduction and contributing to the diminution of acoustic energy. Furthermore, these pores created the necessary space for fiber vibrations during the sound energy-dampening process.

**Figure 8:**
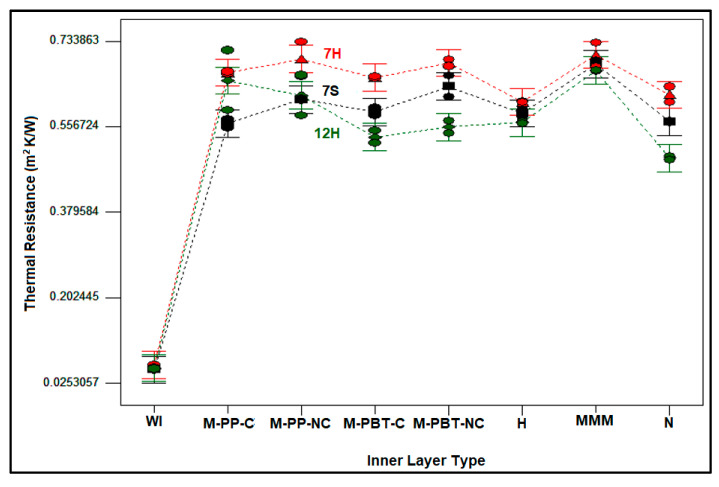
Variation in thermal resistance of the composites by outer and inner layer type.

When considering the impact of the inner layer type on sound insulation, nonwoven composite structures composed of two thermo-bonded layers (without an inner layer) exhibited the lowest noise reduction values for all sound frequencies, meaning that integrating an inner layer improved the sound absorption. Additionally, as indicated in Figure 8, different inner layers were found to be effective at low, moderate, and high sound frequencies. The highest noise reduction values were obtained with composite structures containing calendered single-layer meltblown and hydroentangled inner layers at low sound frequencies. Considering the sound wave equation [9,72], it can be concluded that frequency and wavelength are inversely related, and consequently, sound waves have a greater wavelength at lower frequencies and a shorter wavelength at higher frequencies. More effective bonds are acquired between the fibers constituting the nonwovens in calendered and hydroentangled inner layers due to the effects of heat and pressure for the calendering process and water jet pressure for the hydroentangling process. As a result of the applied processes, the calendered and hydroentangled nonwovens possess a more compact and stronger structure, with a reduced number of air voids inside. It was found that the existence of bonds between the fibers enhanced the material’s resistance to bigger sound waves. Based on the literature, sound absorbers of the resonant type, such as the calendered or hydroentangled layers in our study, were favored over other porous materials for low sound frequency levels [9,12,15,28]. Aside from these results, it can be seen that the composites generated in the study were unfortunately found to be inadequate for sound absorption at low frequencies.

Concerning mid-range sound frequencies, the composite including a three-layered meltblown (MMM) inner layer and 7S fiber-integrated outer layer exhibited the highest noise reduction coefficient value, of approximately 0.90. The composites with an MMM inner layer were followed by calendered and hydroentangled inner layer-inserted counterparts in terms of noise reduction coefficient. This was attributed to the special structure of the MMM nonwoven, which comprises compact resonant-type layers and is thicker due to its multi-layered design compared to other inner layers. At mid-range frequencies, although sound waves decreased in size, the frequency of the sound waves increased, and it was thought that composite structures containing both compact and thicker inner layers, like MMM layers, would be effective in this sound frequency range. Although composites with hydroentangled internal layer exhibited a sound absorption coefficient changing between 76% and 82%, they were unable to outperform the MMM layered counterpart in terms of sound insulation.

The highest noise reduction values in high sound frequency range were obtained with composites composed of non-calendered, nanofiber, and MMM inner layers, respectively. Hydroentangled internal layer-containing composites unfortunately demonstrated lower sound insulation, with a sound absorption coefficient in the 56–64% range, compared to their counterparts. In contrast to low and moderate sound frequencies, the wavelengths shorten and sound waves become denser in number at high sound frequencies. In this context, it was hypothesized that the bulky, thicker, intricate structure with air pores in the non-calendered internal layers would provide an optimal atmosphere for the attenuation of such acoustic waves. Although the splitting action during the hydroentangling process caused the hydroentangled inner layer to have a microfibrous structure, it also led to stronger bonds between fibers, resulting in the lowest thickness among other counterparts. It was assumed that because of its lowest thickness, compact structure, and strong bonds between fibers, the hydroentangled inner layer could not be a solution for this high sound frequency range. Particularly, due to the higher thickness compared to PBT non-calendered counterparts (Table 3), PP-based non-calendered single meltblown-included composites had superior sound absorbing properties. Additionally, an increased number of fibers in the nanofiber inner layer structure was considered to provide higher sound absorption in this sound frequency range. Furthermore, these results were in agreement with previous studies [9,12,15,43,44,45,46,47,48,49,50].

As demonstrated by the figures, composites with nanofiber layers had lower noise reduction coefficients than those with a single meltblown or hydroentangled layer, particularly for low and moderate acoustic frequency levels. On the other hand, composites composed of nanofiber internal layers displayed sound attenuation behavior equivalent to those of single meltblown or hydroentangled alternatives at high acoustic frequency levels. Based on the findings of this study, it can be inferred that composites composed of meltblown or hydroentangled nonwoven layers can impart higher or similar sound insulation properties to composites compared to nanofiber layers. If the faster, more economical, and simpler production of meltblown or hydroentangled nonwovens compared to nanofiber surfaces is considered, it is apparent that utilizing meltblown or hydroentangled layers presents a more beneficial option.

#### 3.2.4. Thermal Resistance

Thermal resistance is frequently a key metric used to evaluate the thermal insulation properties of textile and nonwoven materials, as it quantifies the material’s heat-blocking capacity and is inversely related to thermal conductivity. A higher thermal resistance can be interpreted as an indicator of enhanced insulation performance in nonwoven structures [73,74]. The thermal resistance characteristics of textile products and nonwovens are primarily determined by key parameters such as fiber type, thickness, bulk density, and porosity, all of which significantly influence the volume of air trapped within the materials. Nonwovens with increased volume and a higher content of air voids achieve superior thermal resistance properties, primarily due to the low thermal conductivity of air [72,73]. Additionally, because of the inverse relation between thermal conductivity, nonwoven-containing fibers with lower thermal conductivity exhibit higher thermal resistance. ANOVA results for the thermal resistance of nonwoven composite structures are illustrated in Table 8.

Upon reviewing the ANOVA table, it can be inferred that the best model for describing the variation in thermal resistance of the developed composite structures was the 2FI model, which explained 99.01% of the variation (R^2^ = 99.01%). The types of inner and outer layers individually had a significant effect on the thermal resistance of the nonwoven composites, in addition to the interaction of these factors. Results showed that the inner layer accounted for the largest contribution (94.86%) to the thermal resistance of the composites, suggesting that the thermal resistance of the nonwoven composites is primarily influenced by the properties of this layer.

The effect of the outer layer and inner layer type on the thermal resistance of composites developed in the study is discussed in Figure 8. As indicated from the figure, the thermal resistance values for composites without an inner layer (WI) were the lowest among all composites, and this demonstrates that adding an inner layer had an improving effect on the thermal resistance of the nonwoven composites. Also, the thermal resistances of the composites without an inner layer and different outer layers were found to be closer regardless of the constituent fiber due to the closer porosity values of the outer layers [14].

Despite the low impact of the outer layer, as observed in the ANOVA table, generally the thermal resistances of the composites including the 7H fiber-enriched outer layer was slightly higher than those of the 7S and 12H fiber-containing counterparts. The composites with 7S and 12H fiber-integrated outer layers followed 7H fiber-integrated composites, respectively. Some exceptions observed were believed to result from the regional differences present in all layers constituting the composite. The superior thermal resistance observed in composites with 7H fiber-containing outer layers was attributed to the presence of hollow fibers in the outer layer, as well as a thicker and more voluminous structure compared to other outer layers. Although the fibers in the 12H outer layers also have a hollow cross-section, the composites formed with this external layer exhibited the lowest thermal resistance values. This was linked to the reduced air space within the fiber’s core (Figure 1) and the thinner nonwoven layer structure. It was found that the larger air gaps created by the thicker fiber diameter in the 12H outer layer contributed to thermal loss through the thinner nonwoven structure, ultimately resulting in lower thermal resistance.

When the influence of inner layer types on the thermal resistance of the composites was examined in Figure 8, it could be inferred that the highest thermal resistance values were acquired with nonwoven composites composed of MMM and non-calendered single meltblown inner layers, although the values were closer. As mentioned in Table 5, the raw material of MMM meltblown layer was polypropylene (PP) polymer, and non-calendered meltblown single layers were constituted from either polypropylene (PP) or Polybutylene Terephthalate (PBT) polymer. On the other hand, the nanofiber-based layer was composed of polyamide polymer, and the hydroentangled layer was formed from a 70% polyester/30% polyamide polymer mixture. Based on the available literature, the thermal conductivity of PP polymer varies between 0.17 and 0.22 W/mK [61,75], whereas the thermal conductivity of PBT polymer spans from 0.17 to 0.23 W/mK [76,77]. Furthermore, the thermal conductivity of polyester and polyamide was reported in the literature to be between 0.26–0.27 W/mK and 0.24–0.28 W/mK, respectively [78,79,80,81,82,83]. As observed, the thermal conductivity properties of the polymers constituting the intermediate layers were quite similar, but the raw material for the MMM-based inner layer and single-layer meltblown inner layers, including calendered and non-calendered types, exhibited thermal conductivity properties that were barely any lower. It was hypothesized that the higher thermal resistance of composites with MMM and non-calendered single-layer meltblown intermediate layers could be attributed to the lower thermal conductivity of the base material, as well as structural parameters such as greater thickness and porosity (Table 3). The higher volume of air trapped in the structure of voluminous and thicker non-calendered inner layers led to higher thermal resistance values. Moreover, it was believed that a similar situation was valid for the MMM intermediate layer due to the presence of air voids between the layers despite smaller pore size and the smaller air voids in the MMM inner layer (Table 3). On the contrary, calendered, hydroentangled, and nanofiber-incorporated inner layers resulted in lower thermal resistance values because of smaller pore size, lower thickness, lower porosity, and higher compact structure (Table 3).

Upon evaluating the overall thermal resistance results for the produced multilayered nonwoven composites, it was revealed that the thermal resistance values ranging from 0.49 to 0.73 m^2^K/W were accompanied by thermal conductivity values in the range of 0.043 to 0.063 W/mK. The maximum thermal resistance observed in the formed composites was 0.73 m^2^K/W, which was attained by the composite consisting of a 7H fiber-incorporated outer layer and an MMM inner layer. When the thermal resistance of the composites in this research was compared with the value stated in the Turkish Standard of TS 825 [84] for insulation materials (lower than 0.065 W/mK), it could be concluded that all the obtained composites can be utilized as thermal insulation materials. Moreover, the composites developed in this research may provide a competitive benefit in terms of both thermal conductivity and cost, thanks to the use of recycled PET fiber-based outer layers in comparison with conventional thermal insulators [85,86]. Furthermore, it was believed that using recycled fibers in the majority of the composites contributes to sustainability.

## 4. Conclusions

Based on the experimental investigation exploring the impact of outer and inner layer types on physical and insulation properties of three-layered composites, the significant effects of layer types on selected features of composites were obtained by using statistical analyses conducted by Design Expert 13 software. Moreover, the statistical models employed to clarify the specified properties yielded higher R^2^ values. Regarding the noise reduction for low (100–1000 Hz), moderate (1250–3150 Hz), and high (4000–6300 Hz) sound frequencies and the thermal resistance, the type of inner layer was identified as the most influential factor. Furthermore, the composites in this research demonstrated considerably improved sound absorption coefficients when compared to prior research.

As a continuation of our previous study, this research compared the impact of microfiber-based inner layers, produced by various methods (single-layer meltblown, three-layered meltblown and hydroentangled), on sound and thermal insulation characteristics of the overall composite material. Although inadequate sound absorption values were obtained at low frequencies, a maximum sound absorption coefficient of 0.48 was recorded for the 630 Hz frequency, 0.71 for the 800 Hz frequency, and 0.74 for the 1000 Hz frequency, respectively. Nonwoven composites composed of calendered single-layer meltblown, hydroentangled inner layer, and 7 denier fiber-integrated outer layers exhibited the maximum noise reduction coefficient at low sound frequency levels.

Noise reduction coefficient values ranging from 0.77 to 0.96 were achieved for medium sound frequency levels (1250–3150 Hz), and the highest sound absorption values were obtained with composites constituted with MMM and calendered single-layer meltblown inner layers and outer layers composed of thinner fibers. Additionally, noise reduction coefficient values ranging between 0.94 and 1 were obtained at high sound frequencies (4000–6300 Hz), and non-calendered single-layer meltblown, nanofiber, and MMM nonwovens were determined to be the inner layers that delivered peak noise reduction coefficients. Consequently, it can be inferred that hydroentangled inner layers can be alternatives to calendered single-layer meltblown or nanofiber nonwovens at low sound frequency levels, whereas three-layered meltblown (MMM) layers can be substituted for non-calendered single layers or nanofiber layers at mid and high frequencies. Moreover, considering the rapid, efficient, cost-effective, and simple production processes for single or layered meltblown and hydroentangled nonwovens compared to nanofiber layers, using these layers would undoubtedly offer significant advantages.

Regarding the thermal resistance and conductivity results, all the composites formed in our study can be accepted as thermal insulating materials according to Turkish standards [84]; the maximum thermal resistance and consequently the minimum thermal conductivity values were obtained with the composites with a 7H fiber-integrated outer layer and three-layered meltblown (MMM) or polypropylene-based non-calendered single-layer meltblown inner layers. Furthermore, because their outer layers are produced with recycled PET, these composites exhibited a low-cost advantage compared to market counterparts. Additionally, environmental conservation was supported through the use of sustainable r-PET fibers in the majority of the composites. As a result, considering their sound and thermal insulation capabilities, cost-effectiveness, and eco-friendliness, the produced three-layered nonwoven composites can serve as insulation structures that offer a strategic advantage in relevant applications.

## Figures and Tables

**Table 1:** Layers used within the inner part of the composite.

Layer Code	Manufacturing Process	Image
M-PP-NC	meltblown	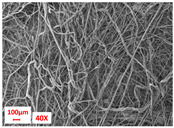
M-PP-C	meltblown and calendering	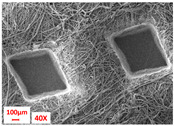
M-PBT-NC	meltblown	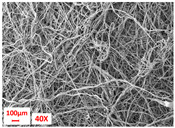
M-PBT-C	meltblown and calendering	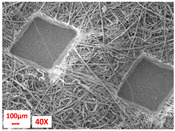
H	spunbond and hydroentangling	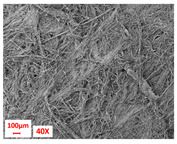
MMM	Meltblown, layering and calendering	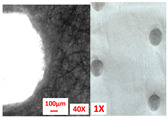
N	Electrospun	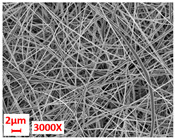

M: Meltblown, PP: Polypropylene, C: Calendered, NC: Non-calendered, PBT: Polybutylene Terephthalate, H: Hydroentangled layer, MMM: Three-layered meltblown layer, N: Nanofiber layer.

**Table 2:** The composite structures formed in the study.

No	Composite Type	No	Composite Type	No	Composite Type
1	[7S]-[M-PP-NC]-[7S]	8	[7H]-[M-PBT-NC]-[7H]	15	[12H]-[H]-[12H]
2	[7H]-[M-PP-NC]-[7H]	9	[12H]-[M-PBT-NC]-[12H]	16	[7S]-[MMM]-[7S]
3	[12H]-[M-PP-NC]-[12H]	10	[7S]-[M-PBT-C]-[7S]	17	[7H]-[MMM]-[7H]
4	[7S]-[M-PP-C]-[7S]	11	[7H]-[M-PBT-C]-[7H]	18	[12H]-[MMM]-[12H]
5	[7H]-[M-PP-C]-[7H]	12	[12H]-[M-PBT-C]-[12H]	19	[7S]-[N]-[7S]
6	[12H]-[M-PP-C]-[12H]	13	[7S]-[H]-[7S]	20	[7H]-[N]-[7H]
7	[7S]-[M-PBT-NC]-[7S]	14	[7H]-[H]-[7H]	21	[12H]-[N]-[12H]

**Table 3:** Internal layer features.

Sample Code	Fiber Fineness (µm)	Weight per Area (g/m^2^)	Thickness (mm)	Calculated Porosity (%)	Pore Size (µm)	Air Permeability (L/m^2^/s)
M-PP-C [14]	4.33 (9.3)	208.4 (3.6)	0.87 (2.5)	73.260	15.0 (4.8)	1.27 (12.6)
M-PP-NC [14]	5.04 (10.3)	210.43 (5.9)	2.36 (3.8)	90.093	31.5 (5.2)	21.06 (5.9)
M-PBT-C [14]	7.69 (11.2)	204.39 (2.3)	0.80 (2.4)	81.123	20.6 (4.4)	6.07 (12.3)
M-PBT-NC [14]	6.11 (8.4)	218.22 (2.9)	1.26 (2.8)	87.144	22.7 (5.9)	13.06 (9.8)
H	6.40 (10.5)	208.5 (7.3)	0.53 (9.8)	70.036	17.1 (4.9)	1.24 (15.8)
MMM	5.33 (8.2)	211.7 (4.6)	2.40 (5.2)	90.207	17.5 (5.3)	8.12 (11.5)
N	0.19 (10.9)	8.3 (3.3)	-	-	0.38 (10.2)	-

Coefficient of Variation (CV%) values are displayed in parenthesis.

**Table 4:** ANOVA Summary for Thickness and Bulk Density of Composite Structures.

ANOVA for Thickness				ANOVA for Bulk Density			
Source	F-Value	*p*-Values	Contribution (%)	Source	F-Value	*p*-Values	Contribution (%)
Model	40.88	<0.0001	96.33	Model	139.06	<0.0001	R^2^ = 98.89
O	156.43	<0.0001	81.93	O	140.29	<0.0001	77.60
I	7.86	0.0006	14.41	I	134.72	<0.0001	21.29
Residual	40.88	<0.0001	3.67	Residual			1.11
Cor Total	156.43	<0.0001	100	Cor Total			100

O: external layer type, I: Internal layer type.

**Table 5:** Analysis of Variance (ANOVA) table for air permeability of composites.

Source	Contribution (%)	Sum of Squares	Degrees of Freedom	Mean Square	F Value	*p* > F	Significance
**Model**	R^2^ = 99.9	237,297.3	23	10,317.27	3987.57	<0.0001	Significant
**O**	0.58	1363.96	2	681.98	263.58	<0.0001	Significant
**I**	98.16	233,167.7	7	33,309.68	12,874.02	<0.0001	Significant
**OI**	1.16	2765.57.	14	197.54	76.35	<0.0001	Significant
**Residual**	0.1	248.39	96	2.59			
**Cor Total**	100	237,545.6	119				

**Table 6:** Variance analysis of the sound absorption coefficient for composites across all sound frequencies.

Source	Contribution (%)	Sum of Squares	Degrees of Freedom	Mean Square	F Value	*p* > F	Significance
**Model**	95.83	49.73	26	1.91	378.88	<0.0001	Significant
**O**	0.11	0.06	2	0.03	5.60	0.0040	Significant
**I**	5.40	2.80	7	0.40	79.25	<0.0001	Significant
**F**	58.24	30.22	1	30.22	5987.22	<0.0001	Significant
**F^2^**	23.57	12.23	1	12.23	2423.41	<0.0001	Significant
**IF**	3.57	1.85	7	0.26	52.43	<0.0001	Significant
**F^3^**	3.25	1.68	1	1.68	333.68	<0.0001	Significant
**IF^2^**	1.69	0.88	7	0.13	24.80	<0.0001	Significant
**Residual**	4.17	2.17	429	0.005			
**Cor Total**	100	51.89	455				

**Table 7:** The variance analysis for low, medium, and high frequency levels.

Low Frequency Levels (100–1000 Hz)				Medium Frequency Levels (1250–3000 Hz)				High Frequency Levels (4000–6300 Hz)			
Source	F Value	*p* > F	Contribution (%)	Source	F Value	*p* > F	Contribution (%)	Source	F Value	*p* > F	Contribution (%)
**Model**	25.90	<0.0001	R^2^ = 94.33	**Model**	58.92	<0.0001	R^2^ = 97.61	**Model**	57.50	< 0.0001	R^2^ = 97.37
**O**	7.50	0.0061	6.07	**O**	3.04	0.0523	2.01	**O**	1.96	0.1783	0.74
**I**	31.15	<0.0001	88.26	**I**	74.19	<0.0001	95.60	**I**	73.37	< 0.0001	96.63
**Residual**			6.67	**Residual**			2.39	**Residual**	-	-	2.63
**Cor Total**			100	**Cor Total**			100	**Cor Total**	-	-	100

**Table 8:** Analysis of variance related to thermal resistance of composites.

Source	Sum of Squares	Degrees of Freedom	Contribution (%)	Mean Square	F Value	*p* > F	Significance
**Model**	1.79	23	R^2^ = 99.01	0.078	104.57	<0.0001	Significant
**O**	0.04	2	2.35	0.021	28.52	<0.0001	Significant
**I**	1.71	7	94.86	0.245	329.17	<0.0001	Significant
**OI**	0.03	14	1.80	0.002	3.13	0.0068	Significant
**Residual**	0.017	24	0.99	0.0007			
**Cor Total**	181	47	100				

## Data Availability

Data are provided in the article.
